# Epidemiological profile of respiratory viruses associated with influenza-like illness and severe acute respiratory infection in Guangzhou, China, 2024–2025

**DOI:** 10.3389/fpubh.2026.1842327

**Published:** 2026-06-19

**Authors:** Pingting Zhu, Mingming Yan, Tengfei Zhou, Qing He, Anna Wang, Xia Tao, Yiyun Chen, Dan Xia, Jingjing Zhang, Aiping Deng, Lan Cao, Yanhui Liu, Xinwei Wu, Pengzhe Qin

**Affiliations:** 1 Guangzhou Center for Disease Control and Prevention (Guangzhou Health and Supervision Institute), Guangzhou, China; 2 Institute of Public Health, Guangzhou Medical University, Guangzhou, China; 3 Guangdong Center for Disease Control and Prevention, Guangzhou, China

**Keywords:** acute respiratory infections (ARIs), epidemiology, influenza-like illness (ILI), post-pandemic era, respiratory pathogens, severe acute respiratory infection (SARI)

## Abstract

**Background:**

The post-pandemic era has brought about marked shifts in the circulation patterns of respiratory pathogens. Nevertheless, comprehensive epidemiological data on acute respiratory infections (ARIs) covering both influenza-like illness (ILI) and severe acute respiratory infection (SARI) remain scarce for southern China. This study aimed to characterize the epidemiological profiles and pathogen spectra of ARIs in Guangzhou, China, from January 2024 to December 2025.

**Methods:**

Respiratory specimens were collected from ILI outpatients and SARI inpatients attending two sentinel hospitals in Guangzhou. All specimens were tested for 23 respiratory pathogens using the NxTAG™ Respiratory Pathogen Panel. Statistical analyses were conducted to explore pathogens distribution stratified by patients’ demographic characteristics.

**Results:**

Among 4,635 ARI cases, the overall pathogen detection rate was 47.96% (*n* = 2,223). Influenza virus (IFV, 14.5%) and human rhinovirus (HRV, 10.1%) were predominant. In ILI cases, IFV (19.1%) and HRV (10.9%) were most common; in SARI cases, HRV (8.5%) and respiratory syncytial virus (RSV, 8.2%) prevailed. Marked age-dependent variations were observed: RSV and bocavirus (BOV) primarily infected preschool children, whereas SARS-CoV-2 and IFV were more commonly detected in adults and older individuals. Seasonally, an earlier IFV peak occurring in December and an out-of-season RSV epidemic in 2025 characterized by a summer peak were observed. Co-infections occurred in 9.76% of positive cases, with IFV/HRV most frequent. Positivity rates were higher in ILI (54.1%) than SARI (34.5%). Logistic regression identified advanced age (≥60 years), RSV in children age < 5 years and MP were associated with SARI.

**Conclusion:**

These findings reveal that the spectrum of respiratory pathogens in Guangzhou is characterized by dynamic patterns of co-circulation and alternating predominance across different time periods and populations. Highlighting the need for age-specific and seasonally tailored interventions and comprehensive diagnostic tools.

## 1 Introduction

ARIs and their progression to SARI pose a persistent global public health challenge, associated with substantial morbidity, mortality, and economic costs (1, 2). Notably, acute lower respiratory infections rank as the fifth leading cause of death worldwide (3). The burden of SARI is particularly high among vulnerable populations, including young children, the older adults, and individuals with chronic cardiopulmonary diseases or impaired immunity. The etiology of ARIs and SARI is complex, involving a diverse spectrum of viral and bacterial pathogens. Their epidemiological patterns—including incidence, severity, and seasonal epidemic peaks—are influenced by multiple factors such as geography, climate, host demographics, and population immunity (4, 5). This complexity highlights the vital significance of continuous pathogen surveillance. Such monitoring is essential for identifying emerging infectious threats, clarifying temporal changes in pathogen circulation, and guiding appropriate clinical management (6).

Previous systematic epidemiological studies on respiratory pathogens across China have predominantly focused on the northern and eastern regions, with the majority of research targeting children under 5 years of age (5–8). A key transportation hub and megacity in South China, Guangzhou features uniquet epidemiological characteristics. Nevertheless, relevant systematic research in this area remain limited. Its typical subtropical monsoon climate, together with high population density and mobility, could drive pathogen circulation patterns distinct from other domestic regions. Therefore, implementing full-scale pathogen surveillance in Guangzhou is vital to clarify ARI epidemiological features in South China and improve the national surveillance system, which can well fill the local data gap.

During the COVID-19 pandemic, the widespread implementation of non-pharmaceutical interventions (NPIs) profoundly altered the traditional transmission dynamics of respiratory pathogens, leading to substantial suppression of certain viruses and disruption of their typical seasonal circulation patterns (59–62). Following China’s comprehensive adjustment of NPIs in December 2022, the country entered a transitional phase in respiratory disease management. In 2023, in response to the complex co-circulation of multiple pathogens, a nationwide multi-pathogen surveillance system was established. In this post-pandemic context, the reshaping of social contact patterns and population immunity may be redefining the baseline epidemiological characteristics of major respiratory pathogens.

Against this backdrop, we conducted a retrospective analysis of population-based surveillance study on ILI and SARI in Guangzhou from 2024 to 2025. This study aims to comprehensively delinate the epidemiological profiles and temporal trends of dominant respiratory pathogen strains under the new normal, and revealed significant associations between specific respiratory pathogens and ARIs across all age groups. Our findings are expected to establish a vital epidemiological baseline for the post-pandemic era and provide a scientific foundation for long-term, evidence-based prevention and control strategies for respiratory infectious diseases.

## 2 Materials and methods

### 2.1 Study design and surveillance

This investigation was coordinated by the Guangzhou Center for Disease Control and Prevention (Guangzhou CDC), and active surveillance of ARIs performed from January 2024 to December 2025. Specimens were collected from two sentinel hospitals in Guangzhou: a municipal children’s medical center and a general tertiary hospital. The pediatric hospital serves as a regional referral hub for childhood illnesses, providing reliable data on pediatric pathogen profiles. Meanwhile, the general tertiary hospital covers a wide population spectrum, including adults, older adults, and severely ill patients, thus enabling comprehensive tracking of epidemiological patterns across age groups.

Prior to the initiation of surveillance, all on-site personnel received standardized training to ensure standardized patient enrollment, specimen processing, and transportation in strict accordance with established protocols. Sentinel hospitals were responsible for case identification, specimen collection, and biological sample transportation, while the reference laboratory of Guangzhou CDC performed all etiological testing. A series of quality control measures were implemented to maintain procedural consistency across sites, including pre-surveillance training, regular on-site supervision, weekly enrollment data summary, routine data validation and annual field inspections.

### 2.2 Case definitions and enrollment criteria

The surveillance targeted both outpatients and inpatients presenting with clinical signs of ARIs. Case definitions were aligned with World Health Organization guidelines (9): ILI was characterized by an acute respiratory infection with documented fever (≥38.0 °C) and either cough or sore throat. SARI was defined as an acute respiratory illness requiring hospitalization, accompanied by a history or presence of fever (≥38.0 °C) and cough, with onset within 10 days. Sampling adhered to the specifications outlined in the National Technical Protocol for Acute Respiratory Infectious Disease Surveillance issued by the Chinese Center for Disease Control and Prevention (China CDC), employing a simple random sampling method for case selection.

Specimens—including throat swabs, nasopharyngeal swabs, nasopharyngeal aspirates, or bronchoalveolar lavage fluid—were collected by trained medical staff on the day of outpatient consultation or within 48 h of admission for hospitalized individuals. Each specimen was immersed in 4 mL of sterile preservation solution and transported to the Guangzhou CDC laboratory together with matched patient data for pathogen detecting. Patients were excluded if they had undergone repeated pathogen testing within a 7-day period yielding consistent positive results or consecutive negative findings.

### 2.3 Pathogen detection and classification

Pathogen detection was performed using a multiplex nucleic acid testing system based on polymerase chain reaction (PCR) combined with bead-based microarray hybridization. The NxTAG™ Respiratory Pathogen Panel (DiaSorin Group, formerly Luminex Corporation, USA) was used in strict accordance with the manufacturer’s protocol. The assay enables the simultaneous detection of 23 respiratory pathogens, including influenza viruses (IFV, covering influenza A and H1, H3, and 2009 pandemic H1N1 subtypes, as well as influenza B), parainfluenza viruses (PIV, types 1–4), *Respiratory syncytial virus* (RSV, subtypes A and B), coronaviruses (COV, types NL63, HKU1, 229E, OC43), *Rhinovirus/Enterovirus* (HRV), *Adenovirus* (ADV), *Human metapneumovirus* (HMPV), *Bocavirus* (BOV), severe acute respiratory syndrome coronavirus 2 (SARS-CoV-2), as well as atypical pathogens such as *Mycoplasma pneumoniae* (MP), *Chlamydia pneumoniae* (CP), and *Legionella* spp. The procedure involves nucleic acid extraction, multiplex PCR amplification, hybridization of amplicons to specific beads, followed by fluorescence signal acquisition using the Luminex MAGPIX^®^ platform and automated interpretation with the accompanying software.

To streamline subsequent statistical analysis, minimize data fragmentation and improve analytical robustness, all viral subtypes, including influenza virus subtypes, parainfluenza virus types 1–4, and respiratory syncytial virus subtypes A and B, were aggregated and grouped at a higher taxonomic level for unified statistical analysis.

### 2.4 Statistical analysis

Data was collected from the surveillance system and stored in Microsoft Excel (Microsoft Corp., Redmond, WA, USA). Continuous variables were presented as medians with inter-quartile ranges (IQRs), and between-groups comparisons were conducted using the non-parametric test (Wilcoxon rank-sum test). Categorical variables were presented as frequencies and percentages. The pathogen detection rate or positivity rate was defined as the proportion of positive samples among all tested samples. Pearson’s chi-square tests or Fisher’s exact tests were used to compare positivity rates between groups. Logistic regression analysis was performed to identify factors associated with SARI, with subgroup analyses stratified by age groups. The results are presented as odds ratio (OR) with 95% confidence interval (*CI*) and *p*-values. To ensure model stability and convergence, pathogens with fewer than 10 positive cases were excluded from multivariable analysis. All statistical tests were two-tailed, and a *p*-value< 0.05 was considered statistically significant. All analyses were performed using R software (version 4.2.2; R Foundation for Statistical Computing, Vienna, Austria), bar charts and line graphs were generated using Microsoft Excel, while heatmaps and forest plots were plotted via R software.

## 3 Results

### 3.1 Demographic characteristics of the study population

A total of 4,635 cases with acute respiratory infection were enrolled in this study, including, 2,590 (55.88%) male and 2,045 (44.12%) female. Participants were categorized into four age subgroups: preschool children aged <5 years (*n* = 1,761,37.99%), school-age children and adolescents aged 5–19 years (*n* = 1,633,35.23%), adults aged 20–59 years (*n* = 820,17.69%), and old adults aged≥60 years(*n* = 421,9.08%). The median age was 6.4 years (IQR: 3–24 years). In terms of case classification, ILI cases were outpatients, accounted for 3,184 (68.69%) cases, whereas SARI cases constituted 1,451 (31.31%).

### 3.2 Overall positivity rates of respiratory pathogens

Of all enrolled patients, 2,223 (47.96%) tested positive for at least one respiratory pathogen. IFV was the most prevalent pathogen (14.50%), followed by HRV (10.12%), RSV (5.80%), SARS-CoV-2 (5.11%), ADV (4.08%), HMPV (4.03%), PIV (3.80%), MP (1.53%), COV (1.47%), BOV (1.38%), and CP (0.17%) (Table 1). Patients in the pathogen-positive group had a significantly lower median age (IQR) than those in the pathogen-negative group (*W* = 2,335,590, *p* < 0.05). No significant difference in detection rates was observed between males (48.19%) and females (47.68%) (*χ*^2^ = 0.118, *p* = 0.731). Age-stratified analysis revealed that preschool children (0–4 years) exhibited the highest positivity rate (52.98%), while the older adults (≥60 years) presented the lowest rate (33.73%), with statistically significant disparities across age groups (*χ*^2^ = 57.075, *p* < 0.05). The overall pathogen positive rate was significantly higher in 2025 compared with 2024 (*χ*^2^ = 34.945, *p* < 0.05). Furthermore, ILI cases showed a significantly higher pathogen detection rate than SARI cases (*χ*^2^ = 152.718, *p* < 0.05) (Table 2).

**Table 1:** Respiratory pathogen detection rates by age group and sex.

Pathogen	Total *N* = 4,635 (%)	<5 years *N* = 1761 (%)	5–19 years *N* = 1,633 (%)	20–59 years *N* = 820 (%)	≥60 years *N* = 421 (%)	Age-*χ*^2^	Age*-p*	Male *N* = 2,590 (%)	Female *N* = 2045 (%)	Gender-*χ*^2^	Gender-*p*
IFV	672 (14.50)	177 (10.05)	275 (16.84)	166 (20.24)	54 (12.83)	58.106	<0.001	374 (14.44)	298 (14.57)	0.016	0.899
RSV	269 (5.80)	189 (10.73)	62 (3.80)	10 (1.22)	8 (1.90)	133.542	<0.001	156 (6.02)	113 (5.53)	0.517	0.472
ADV	189 (4.08)	97 (5.51)	82 (5.02)	7 (0.85)	3 (0.71)	46.912	<0.001	113 (4.36)	76 (3.72)	1.211	0.269
HRV	469 (10.12)	203 (11.53)	180 (11.02)	61 (7.44)	25 (5.94)	29.644	<0.001	271 (10.46)	198 (9.68)	0.767	0.381
PIV	176 (3.80)	103 (5.85)	53 (3.25)	13 (1.59)	7 (1.66)	37.886	<0.001	95 (3.67)	81 (3.96)	0.268	0.604
HMPV	187 (4.03)	98 (5.57)	63 (3.86)	16 (1.95)	10 (2.38)	22.971	<0.001	108 (4.17)	79 (3.86)	0.278	0.598
BOV	64 (1.38)	51 (2.90)	11 (0.67)	1 (0.12)	1 (0.24)	49.274	<0.001	37 (1.43)	27 (1.32)	0.098	0.754
SARS-CoV-2	237 (5.11)	54 (3.07)	70 (4.29)	85 (10.37)	28 (6.65)	66.187	<0.001	116 (4.48)	121 (5.92)	4.871	0.027
COV	68 (1.47)	49 (2.78)	19 (1.16)	27 (3.29)	10 (2.38)	15.014	0.002	59 (2.28)	46 (2.25)	0.004	0.948
MP	71 (1.53)	25 (1.42)	46 (2.82)	0 (0.00)	0 (0.00)	30.740	<0.001	41 (1.58)	30 (1.47)	0.102	0.749
CHL	8 (0.17)	1 (0.06)	4 (0.24)	2 (0.24)	1 (0.24)	2.212	0.530	6 (0.23)	2 (0.10)	—	0.479*

*Fisher’s exact test was used for the comparison of CHL between gender.

**Table 2:** Demographic, seasonal, and classification information of participants with ARIs.

Characteristics	Positive cases *N* = 2,223 (%)	Negative cases *N* = 2,412 (%)	Statistic	*p*
Age, median (IQR)	6 (3 ~ 14)	7 (3.6 ~ 30)	*W* = 2,335,590	<0.001
Gender, *N* (%)				
Male	1,248 (48.19)	1,342 (51.81)	*χ*^2^ = 0.118	0.731
Female	975 (47.68)	1,070 (53.32)		
Age group, *N* (%)				
<5 y	933 (52.98)	828 (47.02)	*χ*^2^ = 57.075	<0.001
5–19 y	787 (48.19)	846 (51.81)		
20–59 y	361 (44.02)	459 (55.98)		
≥60 y	142 (33.73)	279 (66.27)		
Year, *N* (%)				
2024	619 (42.54)	836 (57.56)	*χ*^2^= 24.945	<0.001
2025	1,604 (50.44)	1,576 (49.56)		
Season, *N* (%)				
Spring (Mar–May)	513 (45.56)	613 (54.44)	*χ*^2^ = 8.148	0.043
Summer (Jun–Aug)	602 (50.97)	579 (49.03)		
Autumn (Sep–Nov)	592 (48.64)	625 (51.36)		
Winter (Dec–Feb)	516 (46.44)	595 (53.55)		
Classification of ARIs, *N* (%)				
ILI	1722 (54.08)	1,462 (45.92)	*χ*^2^ = 152.718	<0.001
SARI	501 (34.53)	950 (65.47)		

Age comparisons were conducted using the Wilcoxon rank-sum test, while gender, age group, season and classification of ARIs were analyzed using the chi-square test.

### 3.3 Age-specific distribution patterns of pathogens

Pathogen distribution patterns varied significantly across age groups, revealing prominent age-dependent epidemiology trends. Seven pathogens, including RSV, ADV, HRV, PIV, HMPV and BOV, exhibited consistent age-based distribution characteristics. The detection rates were highest among the preschool children and progressively declined with increasing age. Specifically, RSV showed a detection rate of 10.73% in preschool children, who accounted for 70.26% of all RSV-positive cases. BOV presented a similar epidemiological pattern, with 79.69% of BOV-positive cases occurring in preschool children, whereas its detection rate remained notably low in adults and older adults. PIV and HMPV shared comparable age-specific distribution trends, with the highest detection rates observed in preschool children (PIV: 5.85%; HMPV: 5.57%). Their positivity rates gradually declined in school-aged children and adolescents (PIV: 3.25%; HMPV: 3.86%). Adults exhibited the lowest detection rates, followed by a slight rebound among old adults. For HRV, ADV, and MP, peak detection rates were observed in preschool children and school-aged children and adolescents, who collectively constituted 94.71, 81.66, and 100% of all positive cases for each pathogen, respectively. Notably, MP was undetectable in adult and old adult populations (Table 1).

In contrast, IFV, SARS-CoV-2, and COV exhibited a distinct predominance among adults. The highest detection rates for SARS-CoV-2 were observed in adults (10.37%), followed by old adults (6.65%), school-aged children and adolescents (4.29%), and preschool children (0–4 years) (3.07%). IFV detection rates increased from preschool children (10.05%), peaked in adults (20.24%), and declined in old adults (12.83%) (Table 1)

### 3.4 Seasonal and temporal patterns of respiratory pathogen detection

**Figure 1:**
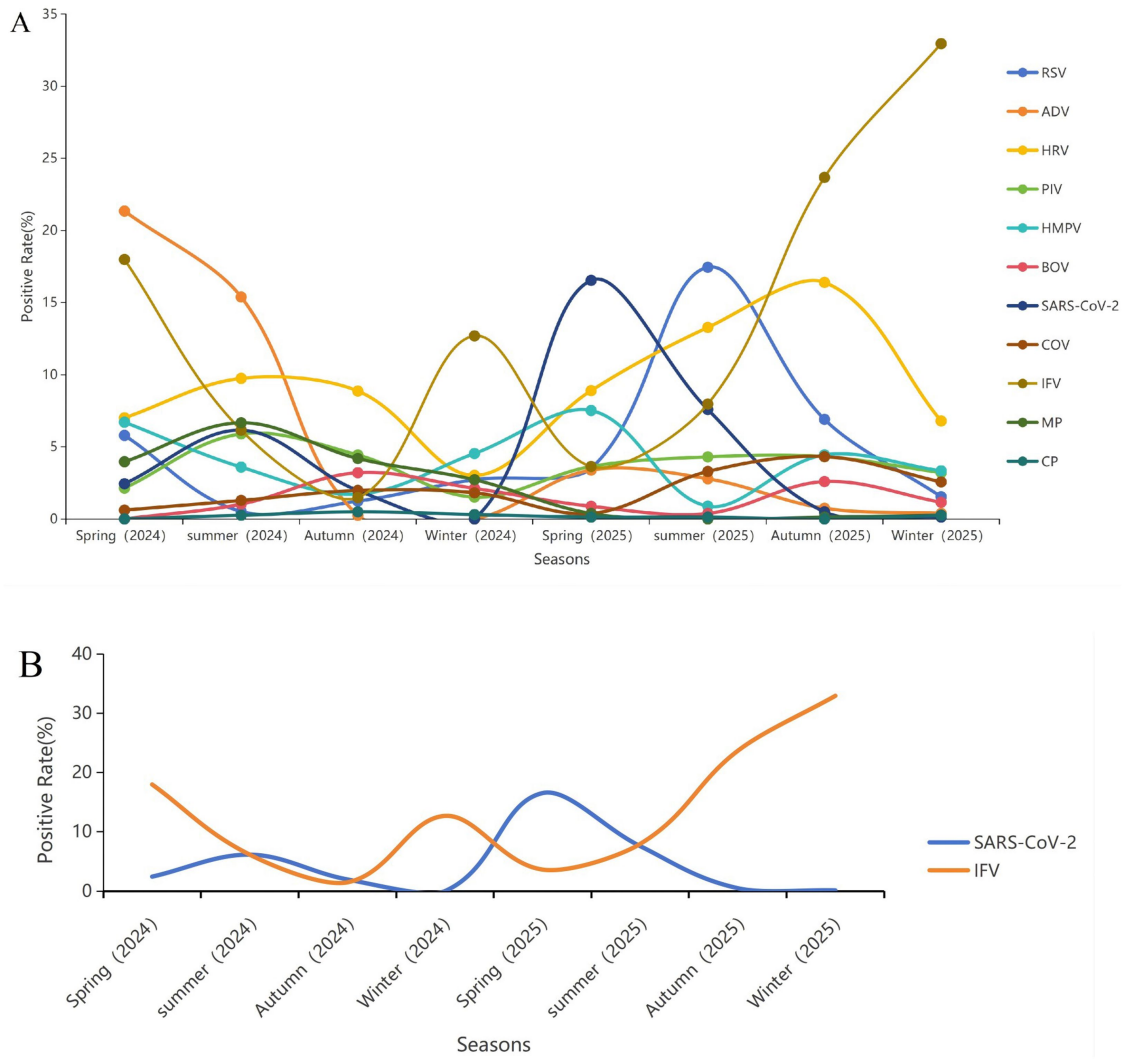
Seasonal distribution of detected respiratory pathogens in Guangzhou, 2024–2025. **(A)** Overall seasonal distribution of all pathogens. **(B)** Seasonal patterns of influenza virus and SARS-CoV-2.

The overall pathogen detection rate of ARIs peaked during the summer (*χ*^2^ = 8.148, *p* = 0.043), with heterogeneous seasonal trends identified among individual viruses (Figure 1A). IFV circulated at high levels during the spring and winter of 2024. In 2025, IFV activity commenced an upward trend in autumn and peaked in December, with a detection rate of 41.67%. SARS-CoV-2 exhibited two discrete epidemic peaks, occurring in summer 2024 and spring in 2025, respectively, showing an antagonistic circulation pattern with IFV. Specifically, when SARS-CoV-2 activity rose to its peak, IFV activity declined to a trough, and vice versa (Figure 1B). RSV exhibited high detection rates during spring and winter 2024, however, its seasonal pattern shifted markedly in 2025, presenting a prominent summer peak and indicating altered seasonal dynamic. ADV and MP maintained relatively high positivity rates in 2024, whereas their detection level declined substantially across all seasons in 2025. HRV exhibited year-round circulation, with a prominent summer peak. Except for January 2024, HRV was persistently detectable, with consistently high positivity observed from June 2025 onward. HMPV and PIV demonstrated relatively stable year-round detections, with epidemic peaks concentrated in winter–spring for HMPV and in summer for PIV. BOV detection rate increased significantly in winter, while COV remained consistently low throughout the year, with its highest detection rate observed in summer (Figure 1A).

### 3.5 The distribution characteristics of co-infections

**Figure 2:**
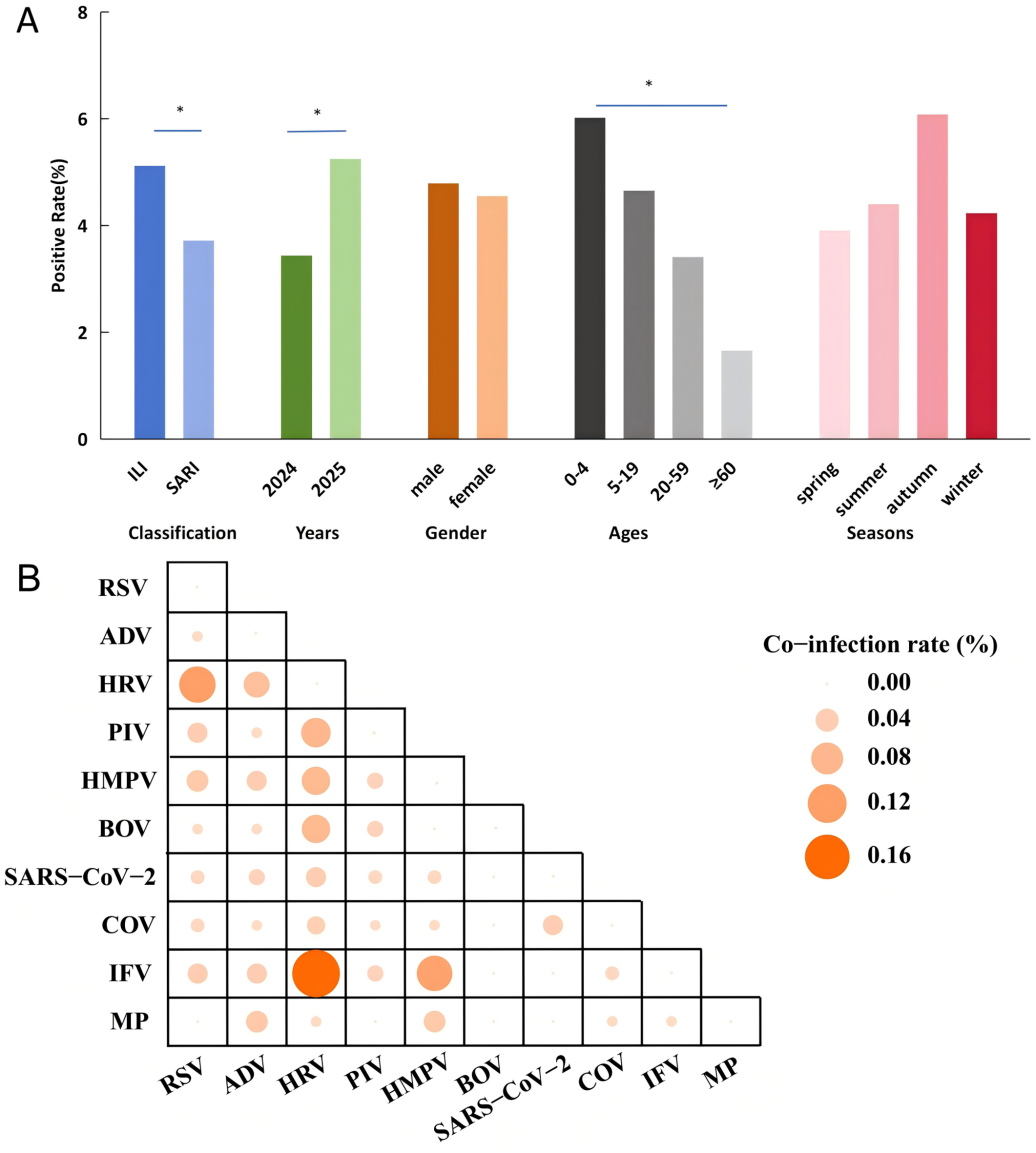
Distribution of co-infections among different groups and combination of dual infections. **(A)** Distribution of co-infections among classifications, years, gender, age groups and seasons. **(B)** Combination of pathogen in dual infections. *indicates statistical significance.

Among the 2,223 positive cases, 217 (9.76%) were identified as co-infection. The co-infection rate was significantly higher in ILI cases (5.12%) than in SARI cases (3.72%), and higher in 2025 (5.25%) than in 2024 (3.44%). Additionally, Age-stratified analysis demonstrated that preschool children (0–4 years) had the highest positivity rate (6.02%), whereas the lowest rates were observed in old adults (≥60 years) (1.66%). All these differences were significant (all *p* < 0.05). No significant differences were observed between gender and among seasons (all *p* > 0.05) (Figure 2A).

Dual infections were the most frequent pattern, constituting 198 cases (94.93% of all co-infections), while triple infections were detected in 11 cases (5.07%). Analysis of pathogen pairs revealed that IFV combined with HRV was the most commonly detected co-infection combination (15.77%), followed by HRV plus RSV (9.60%), IFV plus HMPV (9.09%) (Figure 2B).

### 3.6 Pathogen distribution and associated factors in ILIs and SARIs

The pathogen detection rates for males were 55.17% in ILI cases and 34.80% in SARI cases, whereas females had detection rates of, 52.83 and 34.10%, respectively. Stratified by gender, the pathogen detection rate was significantly higher in ILI cases than in SARI cases (male: *χ*^2^ = 105.134, *p* < 0.05; female: *χ*^2^ = 57.383, *p* < 0.05). Consistently, across all age groups and seasons, the detection rate in ILI cases consistently exceeded that in SARI cases, with statistically significant differences (all *p* < 0.05). Among ILI specimens, IFV showed the highest positivity rate (19.13%), followed by HRV (10.87%) and SARS-CoV-2 (6.66%). In SARI specimens, HRV dominated (8.48%), followed by RSV (8.20%) and IFV (4.34%). ADV, HRV, PIV, SARS-CoV-2, COV, and IFV were significantly more prevalent in ILI cases (all *p* < 0.05). In contrast, MP and RSV demonstrated markedly higher positivity rates in SARI cases (*p* < 0.001). These finds were summarized in Supplementary files.

**Figure 3:**
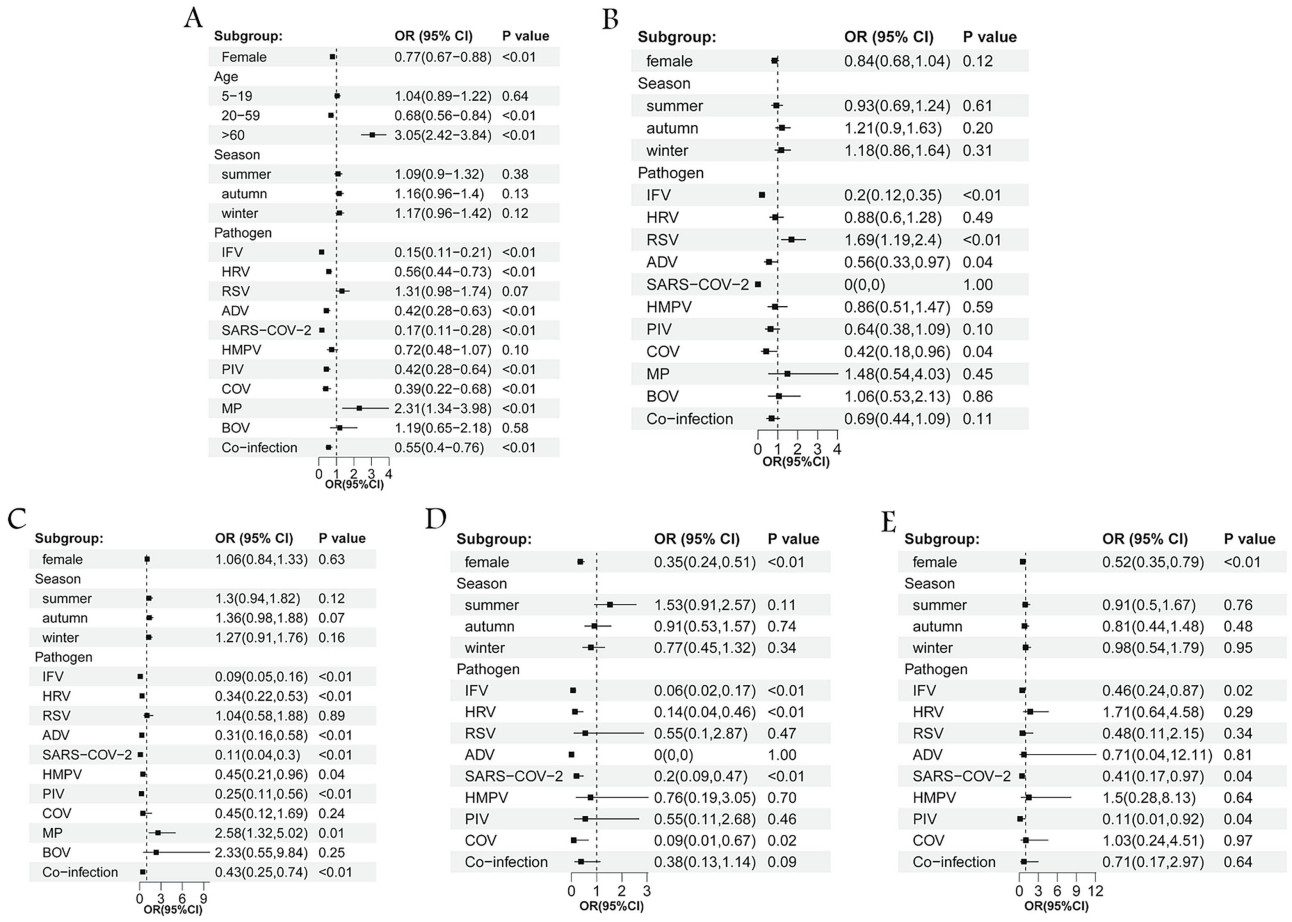
Forest plot of the association between infection with SARI by age groups. **(A)** Among the all population. **(B)** Among the pre-school children (0–4 years). **(C)** Among the school-aged children and adolescents (5–19 years). **(D)** Among the adults (20–59 years). **(E)** Among the older adults (≥60 years).

A binary logistic regression model was constructed with SARI as the dependent variable, and gender, age group, season, and nine pathogens as independent variables. In the all population, logistic regression analysis revealed that male and the older adults were associated with SARI. Compared with patients who tested negative for all pathogens, IFV, HRV, ADV, PIV, COV, and co-infections were associated with ILI, while MP was associated with SARI. Age-stratified analysis showed that among pre-school children, RSV infection was significantly associated with SARI (OR = 1.69, 95% *CI*: 1.19–2.40, *p* < 0.05). A comparable association (OR = 2.58, 95% *CI*: 1.32–5.02, *p* < 0.05) was observed for MP in school-aged children and adolescents. No significant associations between specific pathogens and SARI were observed in the adult or older adults groups (Figure 3).

## 4 Discussion

Acute respiratory infections represent a significant public health and economic burden, underscoring the critical importance of continuous surveillance of pathogen composition and epidemic characteristics for effective prevention and control of respiratory diseases (10). This study systematically analyzed the age-specific distribution and seasonal epidemic patterns of respiratory pathogens in Guangzhou during 2024–2025. The overall pathogen detection rate in our study was 47.96%, which is comparable to previously documented in Rome (46.4%) (11) and Yongzhou (43.12%), China (12). The positivity rate lower than the pre-pandemic rate reported in Shanghai (78.9%), an eastern Chinese city metropolis (13), yet higher than that recorded in Guizhou (39.57%), a western Chinese region (63). The co-infection detection rate in our study was 9.76%, higher than that reported in Shijiazhuang (8.9%) (5) but lower than that reported in Chongqing (15.1%) (14). Potential reasons for these differences may include variations in the range of pathogens tested and the test methods used as well as differences in the demographic composition of the study populations.

All targeted respiratory pathogens were successfully detected in our study, demonstrating a complex and diverse pathogens spectrum in the local area. Overall, IFV, HRV, SARS-CoV-2, and RSV were the most frequently detected pathogens. This pathogen profile partially diverges from those documented in other regions (11, 15–17), highlighting geographical variations in pathogen distribution. Notably, IFV and HRV were predominant in both ILI and SARI cases, which align with previous observations from the United States (18) and Fujian Province, China (19). Existing evidence indicates that HRV not only cause common colds but can also independently or jointly with other pathogens lead to pneumonia, underscoring their potential role in SARI (20, 21).

Numerous studies have documented marked age-based disparities in respiratory pathogen distribution (22, 23). Consistent with a national ARI surveillance study in China (24) and a community-based investigation conducted in Shanghai (8), our results demonstrated that preschool children aged exhibited the highest viral detection rate, followed by a gradual declining trend with advancing age, while older adults presented the lowest detection rate. The observed pattern may be attributed to the immature immune system in young children, higher exposure risks in collective settings such as childcare facilities and schools, as well as the progressive development of immunity with age, which enhances resistance to infections (19, 25, 26).

Furthermore, our study identified IFV, RSV, and HRV as the predominant pathogens among preschool children under 5 years, aligning with multiple previous epidemiological findings (22, 27). In contrast, SARS-CoV-2, IFV, and HRV dominated in adult and older adult population, consistent with earlier studies (12, 27–30). These observations indicate a higher susceptibility to SARS-CoV-2 infection in advanced age groups. Although the precise mechanisms underlying such age-dependent disparities remain incompletely elucidated, existing evidence suggests that delayed innate immune responses and reduced adaptive immune function in adults and the older adults may impair viral clearance and prolong viral replication, thereby increasing susceptibility to symptomatic and persistent infection (29, 31). Additionally, this population more likely to be identified and captured by surveillance systems.

By comparison, MP and ADV exhibited the highest detection rates among school-aged children and adolescents, while their positivity rates remained low in adults and older adults. This distribution pattern corresponds with surveillance data documented across China (24), the United States (22), and as well as England and Wales (32). Collectively, these results emphasize that age constitutes a critical determinant shaping the spectrum of respiratory pathogens. Accordingly, healthcare facilities should therefore strengthen pathogen identification and optimize clinical management of age-specific respiratory diseases.

Seasonality is widely recognized as a key feature of most respiratory virus infections. Multiple surveillance reports have documented that NPIs implemented during COVID-19 lockdowns disrupted the typical circulation patterns of many pathogens (33). In the current post-pandemic phase, with the resumption of social activities and the relaxation NPIs, the transmission dynamics of respiratory pathogens are undergoing readjustment and rebalancing. Our preliminary observations indicate that the IFV activity peaked in December, consistent with the contemporaneous national surveillance data (64). However, this pattern differs from the pre-pandemic surveillance reports, which typically documented a winter peak around February (34). The observed shift suggests that the seasonal peak of IFV in the post-pandemic era may have advanced and that its epidemic timing remains in a state of dynamic transition. HRV, COV, and SARS-CoV-2 showed higher detection rates in summer, whereas PIV and HMPV showed stable year-round circulation, which is consistent with observations reported from Fujian Province, China (6). ADV and MP exhibited relatively high detection rates in 2024 but decreased to low levels throughout 2025. This may be attributed to the substantial outbreaks of ADV and MP that occurred in Guangzhou and many other parts of China during 2023–2024 (35, 36), which likely led to elevated population immunity against these pathogens, resulting in lower circulation in 2025. Notably, our surveillance data show that the epidemic peak of RSV in 2025 occurred during the summer months (June–August), whereas previous studies in Guangzhou reported that RSV activity primarily peaked in winter–spring season (37). This out-of-season epidemic pattern is consistent with the epidemiological shifts observed in other regions of China during and after the pandemic (37–39). These findings suggest that the seasonal peak of RSV may continue to shift in the coming years, further underscoring the importance of sustaining surveillance in the post-pandemic era. In addition, we observed an alternating circulation between SARS-CoV-2 and IFV, characterized by a “seesaw effect” (40). The sequential emergence of epidemic peaks, each lasting from several weeks to months, mirrors the see-saw dynamics reported globally and highlights the asynchronous and potentially competitive circulation of SARS-CoV-2 and influenza viruses in the post-pandemic era (41).

Currently, comparative studies on pathogen profiles between ILI and SARI cases remain limited. Across sexes, age groups, and seasons, the pathogen detection rate was consistently higher in ILI than in SARI. These results are consistent with reports from several countries (17, 34). However, the ILI and SARI case definitions were developed for influenza surveillance and rely primarily on clinical symptoms (9). ILI cases are identified in outpatient, with sampling typically occurring early in illness, facilitating pathogen detection. In contrast, SARI affects hospitalized populations, many patients may have received treatment before sampling, potentially suppressing or clearing pathogens and hindering pathogen detection. Additionally, the sensitivity of pathogen detection varies significantly by specimen type. A paired comparison of throat and nasopharyngeal swabs in Kenya demonstrated that among SARI patients, throat swabs were less likely than nasopharyngeal swabs to yield influenza B virus, PIV-2 and PIV-3 but more likely to yield adenovirus and influenza A virus (17, 42). Notably, a potential limitation to the detection of respiratory viruses in our study was that throat swabs were predominantly used. Such biases may lead to a lower observed pathogen detection rate in SARI cases than in ILI cases.

Logistic regression analysis identified significant associations between specific respiratory pathogens and ARIs. In the full-age cohort, positive detection of MP and age over 60 years were significantly associated with SARI, indicating that older adults had a relatively higher risk of developing SARI. This may be attributed to age-related declines in physiological function, weakened immunity, and an increased prevalence of underlying comorbidities, all of which may render the older adults more susceptible to severe illness (43–45). Association between pathogens and SARI was analyzed through age-stratified analysis. Children aged 0–4 years, positive detection of RSV was linked to SARI, indicate that RSV infection increases the likelihood of children developing SARI, which is consistent with previous studies identifying RSV as a risk factor for lower respiratory tract infections in children (20, 27). Severe RSV infection involves an exacerbated host inflammatory response, leading to airway narrowing and bronchiolitis in infants, which frequently progresses to pneumonia (20). Thus, when RSV infection is identified in a child, clinicians should maintain a high index of suspicion for possible progression to SARI and implement timely interventions. In school-aged children and adolescents, MP was significantly associated with SARI. This aligns with previous reports indicating a high prevalence of MP infection in this age group, where it primarily manifests as acute lower respiratory tract infection (46). As a symptom-based surveillance definition (47), ILI has a diverse etiology. Beyond IFV as the classic pathogen, numerous studies have confirmed that HRV and PIV are also common causative agents of ILI (47, 48). This aligns with our findings that positive IFV, HRV, ADV, PIV detection correlates with ILI. In our study, a negative association was observed between co-infections and SARI, which is consistent with findings from a study conducted in Zhejiang China on the risk factors between co-infections and pneumonia (27). The co-infection patterns identified in our study were predominantly virus-virus combinations. A plausible hypothesis is that virus-induced interferons and other cytokines may generate cross-protective effects, potentially contributing to this association (49–51). However, potential confounding factors such as comorbidities and vaccination status were not addressed in our logistic regression analysis, which may influence the risk of progression to SARI among patients with acute respiratory infections. Detailed clinical histories and vaccination status should be collected to better estimate the association between pathogens and SARI risk in future research.

Multiple viruses can infect the same host, leading to respiratory viral co-infections, which occur when two or more respiratory viruses are simultaneously present in an individual. In our study, preschool children harbored the highest proportion of co-infections, and comparable results have been documented in previous studies (52). An analysis of the prevalence of mono-infections and co-infections of 13 respiratory viruses over 5 years (2013–2018) indicated that respiratory viral co-infections are more predominant in children, especially those under 5 years of age (53). This may be partly explained by the immature immune system of preschool children, which could result in weaker defense capabilities against multiple pathogens (52, 54). This finding suggests that respiratory infection prevention and control strategies for preschool children should shift to a “multi-pathogen co-prevention” approach to address the complex situation of co-circulating respiratory pathogens in the post-pandemic era. IFV/HRV was the most commonly detected co-infection combination in our study, followed by HRV/RSV and IFV/HMPV. A analysis from German university hospitals, indicated that the combination of HRV/BoV exhibited the highest co-infection rate (55). Some reports indicate that RSV frequently co-infects with ADV (56), BOV (57), and IFV (58) these suggested viral co-infection combinations may vary across geographical regions. Thus, monitoring the prevalence of different pathogen co-infection combinations can help provide targeted prevention and control measures through surveillance.

Several limitations of this study should be acknowledged. First, the representativeness of the study population is constrained by the use of only two sentinel hospitals, with an apparent overrepresentation of pediatric cases, which may introduce selection bias and limit generalizability of our findings. Second, the overall detection rates of BOV and CP were relatively low. Constrained by the current sample size and surveillance period, these low detection rates may limit the analysis of epidemiological trends and subgroup comparisons. Third, comprehensive clinical data, including comorbidities and vaccination status, were unavailable. Potential confounding factors were not addressed, which prevented the assessment of the association between these factors and pathogen positivity or disease severity. Fourth, the coverage of pathogens was incomplete. Although we tested for common respiratory viruses and atypical pathogens, other important bacterial pathogens, such as *Haemophilus influenzae*, *Klebsiella pneumoniae*, and *Streptococcus pneumoniae*, were not included. This may have led to under detection of viral-bacterial co-infections and precluded a comprehensive assessment of the role of bacterial pathogens in respiratory infections.

In conclusion, this study characterized the epidemiological features of respiratory pathogens circulating in Guangzhou during 2024–2025. The overall detection rate was 47.96%, with IFV, HRV, SARS-CoV-2, and RSV identified as the predominant pathogens. Marked age-dependent variations was observed: RSV and BOV primarily infected preschool children, whereas SARS-CoV-2 and IFV were more commonly detected in adults and older individuals. With regard to Seasonality, we observed post-pandemic shift in circulation patterns, including an earlier IFV peak occurring in December and an out-of-season RSV epidemic peak in 2025 as well as an alternating circulation between SARS-CoV-2 and IFV. Pathogen detection was higher among ILI cases than among SARI cases, with advanced age, MP and pediatric RSV infection were significantly associated with SARI. These findings establish an important epidemiological baseline for respiratory pathogens in southern China. Future multicenter studies with incorporating broader pathogen detecting are warranted.

## Data Availability

The original contributions presented in the study are included in the article/Supplementary material, further inquiries can be directed to the corresponding authors.
