# Supplementary material for: Epidemiological profile of respiratory viruses associated with influenza-like illness and severe acute respiratory infection in Guangzhou, China, 2024–2025
Source: Front Public Health. 2026 Jun 19;14:1842327. doi: 10.3389/fpubh.2026.1842327 (PMC13330106; doi:10.3389/fpubh.2026.1842327)
Supplement: Supplementary file 1 [file Table_1.DOCX]

**Supplementary file**

| Distribution of pathogens detected positive by ILI and SARI | | | | |
| --- | --- | --- | --- | --- |
| Characteristics | ILI N= 3184(%) | SARI N=1451(%) | χ2 | P |
| Gender |  |  |  |  |
| Male | 939(55.17%) | 309(34.80%) | 105.134 | < 0.001 |
| Female | 783(52.83%) | 192(34.10%) | 57.383 | < 0.001 |
| Age group |  |  |  |  |
| 0-4years | 675(55.69%) | 258(46.99%) | 11.477 | 0.001 |
| 5-19years | 638(56.21%) | 149(29.92%) | 95.831 | < 0.001 |
| 20-59years | 335(51.54%) | 26(15.29%) | 71.835 | < 0.001 |
| ≥60years | 74(39.57%) | 68(29.06%) | 5.139 | 0.023 |
| Season |  |  |  |  |
| Spring (Mar - May) | 416(52.13%) | 97(29.57%) | 47.688 | < 0.001 |
| Summer (Jun - Aug) | 452(56.64%) | 150(39.16%) | 31.631 | < 0.001 |
| Autumn (Sep - Nov) | 450(54.95%) | 142(35.68%) | 39.799 | < 0.001 |
| Winter (Dec - Feb) | 404(52.54%) | 112(32.75%) | 37.262 | < 0.001 |
| Pathogen |  |  |  |  |
| RSV | 150(4.71%) | 119(8.20%) | 22.21 | < 0.001 |
| ADV | 148(4.65%) | 41(2.83%) | 8.465 | 0.004 |
| HRV | 346(10.87%) | 123(8.48%) | 6.26 | 0.012 |
| PIV | 136(4.27%) | 40(2.76%) | 6.26 | 0.012 |
| HMPV | 138(4.33%) | 49(3.38%) | 0.048 | 0.827 |
| BOV | 39(1.22%) | 25(1.72%) | 1.816 | 0.178 |
| SARS-CoV-2 | 212(6.66%) | 25(1.72%) | 50.04 | < 0.001 |
| COV | 83(2.61%) | 22(1.52%) | 5.355 | 0.021 |
| IFV | 609(19.13%) | 63(4.34%) | 175.769 | < 0.001 |
| MP | 28(0.88%) | 43(2.96%) | 28.702 | < 0.001 |
| CHL* | 4(0.13%) | 4(0.28%) | - | 0.268 |

Note:* refers to Fisher’s exact test
